# Visual and cognitive functioning among older adults with low vision before vision rehabilitation: A pilot study

**DOI:** 10.3389/fpsyg.2023.1058951

**Published:** 2023-03-22

**Authors:** Gabrielle Aubin, Natalie Phillips, Atul Jaiswal, Aaron Paul Johnson, Sven Joubert, Vanessa Bachir, Eva Kehayia, Walter Wittich

**Affiliations:** ^1^School of Optometry, Université de Montréal, Montréal, QC, Canada; ^2^Department of Psychology, Concordia University, Montréal, QC, Canada; ^3^Department of Psychology, Université de Montréal, Montréal, QC, Canada; ^4^School of Physical and Occupational Therapy, McGill University, Montréal, QC, Canada

**Keywords:** dementia, macular degeneration, aging, vision rehabilitation, visual impairment, low vision (re)habilitation

## Abstract

**Introduction:**

The occurrence of age-related vision changes is inevitable. However, some of these changes can become pathological. Research indicates that vision and hearing loss is correlated with age-related cognitive decline, and with a higher risk of developing dementia due to Alzheimer’s disease. Low vision rehabilitation could possibly be a protective factor against cognitive decline, as it provides the clients with compensatory strategies to overcome their visual deficits.

**Objectives and hypothesis:**

The aim of this pilot study was to assess correlations between visual and cognitive functions in older adults referred for low vision rehabilitation. We hypothesized that more severe impairment of visual acuity and contrast sensitivity would be correlated with more advanced levels of cognitive impairment. The second objective was to examine which of these correlations would remain significant once established variables that influence cognition are statistically removed (e.g., age, education).

**Methods:**

Thirty-eight older adults (age range: 66–97 years old) with a visual impairment (acuity <20/70) were recruited before the onset of their low vision rehabilitation. They underwent vision (reading acuity, reading speed, contrast sensitivity), hearing (audiogram, speech-in-noise perception) and cognitive (global cognition, memory, executive functions) testing, and demographic information was obtained.

**Results and discussion:**

Correlations among global cognition and visual aid use, memory and reading speed, memory and contrast sensitivity, memory, and visual aid use, and between executive functions and contrast sensitivity were significant. Correlations between contrast sensitivity and memory, as well as between global cognition and visual aid use remained significant after controlling for age and education. The present study is relevant to clinicians who are assessing the cognitive status of older adults, such as neuropsychologists, because it highlights the importance of considering low vision when administering neuropsychological tests, especially to persons who have not yet received rehabilitation for their visual impairment.

## Introduction

Changes in vision, such as the inevitable arrival of the need for reading glasses in middle age (presbycusis) are expected as a part of the normal aging process (Haegerstrom-Portnoy et al., 2002); however, there are eye diseases associated with aging that can further impair the visual abilities of older adults (Wong et al., 2014). Some visual functions change as a result of visual pathologies, such as age-related macular degeneration (AMD), glaucoma or diabetic retinopathy, all of which can cause low vision (Wittich and Gagné, 2016). Low vision (LV) has been described as a decline in visual functions that alters the ability to complete visual tasks, and that cannot be treated with corrective or contact lenses, or other surgical or medical interventions (Corn and Erin, 2010). The World Health Organization (2019) predicts that the number of individuals with AMD will increase 1.2-fold from 2020 (roughly 195 million) to 2030 (roughly 243 million). Interestingly, research indicates that visual decline due to AMD, and vision loss in general, are correlated with age-related cognitive decline, and persons with AMD are at higher risk of developing dementia due to Alzheimer’s disease (Rogers and Langa, 2010; Zhou et al., 2016; Nagarajan et al., 2022). The link between visual and cognitive functioning and aging has a long-standing knowledge base (Lindenberger and Baltes, 1994; Lindenberger et al., 2001); however, the majority of correlational explorations of vision and cognition have been conducted with population-based data sets (Hong et al., 2016) or have focused on specific sub-populations with cognitive or visual diagnoses [e.g., vision in patients with Alzheimer’s disease (AD; Kirby et al., 2010), or cognition in persons with AMD (Zhou et al., 2016)] that have received treatment or are being treated. Low vision rehabilitation is a service that aims at improving daily living by providing techniques to use remaining intact vision. We are unaware of any studies on the effect of low vision rehabilitation on cognition that have been able to include participants with low vision (e.g., visual acuity below 20/60) that had not (yet) received rehabilitation services. A recent longitudinal study focused on functional improvement in activities of daily living in veterans with low vision, with or without cognitive impairment, but the study did not assess cognition pre- and post-rehabilitation (Whitson et al., 2020). The present pilot study aimed to explore the relationship among sensory and cognitive variables, specifically in older adults who had not yet received low vision rehabilitation, thereby presenting an opportunity to better understand the relationship among their pre-rehabilitation sensory-cognitive abilities. This understanding is required for future studies that will explore the effect vision rehabilitation may have on cognition in older adults with low vision (Wittich et al., 2021).

As a result of the normal aging of the visual system, near visual acuity is expected to decrease somewhat, making the reading of small print more challenging (Pierscionek and Weale, 1995). Similarly, aging affects cognitive abilities, such as memory and processing speed (Park and Gutchess, 2016), and a decline in cognitive function is expected. In addition, cognitive pathologies, such as AD and vascular dementia, are two of the most predominant forms of dementia, causing significant changes in cognition (World Health Organization, 2017).

Several studies have linked changes in visual function (e.g., visual acuity, reading acuity) to changes in cognitive function (Rogers and Langa, 2010; Dupuis et al., 2014; Zhou et al., 2016; Davies-Kershaw et al., 2018; Zheng et al., 2018; Ehrlich et al., 2021). For example, cognitive impairment, as measured by on a score of lower than 28 on the Mini Mental State Examination (MMSE), is related to slower reading speed on the Minnesota Low Vision Reading Test (MNRead; Swenor et al., 2016). Similarly, impairment in visual acuity has been linked to lower scores on the Montreal Cognitive Assessment (MoCA; Dupuis et al., 2014), a screening tool designed to identify persons at risk for mild cognitive impairment. Additionally, individuals with mild cognitive impairment and AD have higher rates of reduced reading acuity than individuals with subjective cognitive decline (Wittich et al., 2019). In a meta-analysis, the presence of AMD has been linked to lower scores on various cognitive tests (i.e., Mini-Mental State Examination, Mini-Cog test, Trail Making Test A; Zhou et al., 2016).

Several studies have found vision-related abnormalities in individuals with AD. Visual deficits (e.g., difficulty reading, loss of contrast sensitivity) are commonly found in AD (Lee and Martin, 2004), and visual complaints are often reported by AD patients in the early stages of the disease and may precede the memory decline (Ikram et al., 2012). In addition, a decrease in the visual field and/or contrast sensitivity, as well as fixation problems, are common in AD patients (Ikram et al., 2012). A longitudinal study found that self-reported low vision was a risk factor for AD (Davies-Kershaw et al., 2018). Moreover, this relationship between AD and visual deficits is also present in the brain at the structural level, whereby the topological organization of higher-level visual networks is altered in individuals with AD and mild cognitive impairment (Deng et al., 2016).

Furthermore, several studies focusing on functional outcomes explored the link between changes in vision (e.g., visual acuity, contrast sensitivity) and changes in cognition (e.g., global cognition, executive functions, psychomotor speed). Longitudinal studies have demonstrated an association between low vision and cognitive decline over time, whereby the severity of the decline in visual acuity over 8 years, as measured by the ETDRS chart (Early Treatment of Diabetic Retinopathy Study), is linked to the severity of the cognitive decline at the 8-year follow-up, as measured by the MMSE (Zheng et al., 2018). Another study showed that low vision is associated with the transition from normal cognition to cognitive impairment or dementia, using Digit Symbol Substitution Test and the Modified MMSE), to measure cognition (Ehrlich et al., 2021). A longitudinal study assessing multiple aspects of visual functioning (e.g., contrast sensitivity, visual acuity, and stereo acuity) found that impairments in these measures of vision were associated with increased risks of a decrease in psychomotor speed, worse executive functioning, and general cognitive decline over 9 years (Swenor et al., 2019). A risk factor for cognitive decline and AD is uncorrected self-reported low vision (Rogers and Langa, 2010), while wearing glasses and having good visual acuity can be protective against cognitive decline (Spierer et al., 2016).

Many mechanisms have been postulated to explain this link between sensory and cognitive decline. First, the *information degradation hypothesis* states that neurobiological processes (e.g., AMD) weaken the strength of the perceptual signal, which, in turn, negatively affect perceptual and cognitive processing (Monge and Madden, 2016). In the case of AMD, the visual decline may cause a decrease in the stimulation, thereby resulting in cognitive decline. Second, the *sensory deprivation hypothesis* states that a persistent absence of adequate sensory input will create neuronal atrophy and will later result in cognitive decline (Clay et al., 2009). In the case of AMD, the reduced visual input due to visual decline may create neuronal atrophy, meaning that cognitive decline may result later because of AMD. Third, the *cognitive compensation hypothesis* states that cognitive functions could be used to compensate for the visual decline, therefore monopolizing the cognitive resources and causing cognitive decline (Roberts and Allen, 2016). In the case of AMD, this could mean that cognitive resources, such as attention, could be used to compensate for vision loss. Therefore, not all cognitive functions are available for higher-level tasks. For example, as attention is a key function in the encoding process, this compensation could result in less in-depth encoding, leading to memory decline.

Another hypothesis is the common cause hypothesis, which states changes in sensory and cognitive ability are due to a single common cause, such as the aging brain (Kiely and Anstey, 2015). Other hypotheses have also been proposed to explain the relationship between concomitant changes in sensory and cognitive functions. Social participation has been proposed as a key factor in cognitive health. For example, individuals with vision loss might fear falls when going outside and prefer not to attend social event for this reason. This reduced participation might be followed by a reduction in cognitive capacities (Bourassa et al., 2015).

Recently, some anatomical studies have linked AMD and AD (Dentchev et al., 2003; Chang et al., 2014; Ratnayaka et al., 2015). The brain and the retina share many similarities, such as the same embryological origin, as well as some anatomical and physiological features (Ikram et al., 2012). Chang et al. (2014) presented evidence that the three well-known AD biomarkers—protein amyloid-ß, senile plaques, and neurofibrillary tangles, are also present in the retina of AD patients. Another study linked amyloid-ß deposits in the retina to AMD (Dentchev et al., 2003). These discoveries are an important step in understanding the critical link between AMD and AD.

According to the first three hypotheses, the degradation of the sensory input is proposed to be linked to cognitive decline. The first two hypotheses state that the degradation of the sensory input is causing the degradation of cognition. Therefore, focusing on ways to reinstate the visual input in AMD patients could theoretically preserve or potentially improve cognitive functioning. According to the third hypothesis, reducing the effort needed to see and read could reduce the cognitive demands for tasks involving reading, and more cognitive resources could be allocated to higher-level tasks. Therefore, in line with all three hypotheses, low vision rehabilitation should be a way to preserve or even improve cognitive functioning in patients with AMD. At present, little is known about any relationship among sensory and cognitive variables before the vision rehabilitation process in persons living with AMD.

In Quebec, individuals with vision loss have access to rehabilitation services and assistive devices provided by the government at no cost to the client (Government of Quebec, 2022). Eligible individuals can undergo low vision rehabilitation to maximize their residual vision and learn strategies to compensate for their vision loss (Gordon et al., 2015). These services can include adaptations of the client’s physical environment to ensure proper lighting and adaptive techniques for reading, instruction in the use of assistive devices to facilitate visual activities, and other considerations that might be appropriate for the client. Low vision rehabilitation has been linked to an improvement in quality of life and participation in activities of daily living (Binns et al., 2012). However, to our knowledge, there are currently no data available that evaluate the impact of low vision rehabilitation on cognition in older adults.

### Pilot study objectives

The first objective of this pilot study was to assess correlations between the sensory and cognitive functions in older adults referred for low vision rehabilitation. We hypothesized that more severe impairment of visual acuity and contrast sensitivity would be correlated with more advanced levels of cognitive impairment (e.g., processing speed, executive functioning, and memory). Should such relationships exist, the second objective was to examine which of these correlations would remain significant once established variables that influence cognition are statistically removed (e.g., age, education).

## Methodology

The Institutional Review Board approval for this study was obtained from the *Centre de recherche interdisciplinaire en réadaptation du Montréal métropolitain* (CRIR #1284-1217). Additional details about the larger protocol context, within which these pilot data were collected, have been published elsewhere (Wittich et al., 2021).

### Participants

Low vision participants were recruited from the two rehabilitation centres in the Montreal region (*Centre de Réadaptation Lethbridge-Layton-Mackay du CIUSSS du Centre-Ouest-de-l’Île-de-Montréal*, and *l’Institut Nazareth et Louis-Braille du CISSS de la Montérégie-Centre*). Inclusion criteria were: being at least 65 years old, having a diagnosis of AMD as confirmed by the referring optometrist or ophthalmologist, and being eligible for low vision rehabilitation according to the following Quebec Health Ministry criteria (Government of Quebec, 2022): visual acuity in the better eye of < 20/70 (6/21; ETDRS or Feinbloom) with standard optical correction of lesser than four dioptres, or ≤ 20/60 (6/18) for individuals with a degenerative visual problem and perception of impairment. Exclusion criteria were having a neurological disorder as noted in the patient file or self-reported by the patient, a severe hearing loss (> 70 dB mean pure-tone average hearing level in the better ear), or a diagnosed form of dementia, as noted in the participants’ rehabilitation file or referral. The sample was composed of 38 participants (age range: 66–97 years old). Their demographic information can be found in Table 1.

**Table 1:** Demographic table—frequencies.

Demographic variable	Sample (*n* = 38)
*Age*	
65–75	8
76–85	9
86+	20
*M*(SD)	83,3(8,5)
*Sex (%)*	
Female	23(60,5)
Male	15(39,5)
*Education (%)*	
Elementary school	9(23,7)
High school	11(28,9)
College	8(21,1)
University bachelor	7(18,4)
University master/PhD	3(7,9)
*Langage (%)*	
English	20(52,6)
French	18(47,4)
*DASS (M(SD))*	
Depression	6,6(7,7)
Anxiety	5,1(4,9)
Stress	7,5(6,3)
Total	19,2(15,8)

DASS: depression, anxiety and stress scale. M: mean; SD: standard deviation.

## Materials

All tests were selected because they are suited for individuals with vision impairment (i.e., they do not depend on vision), are available and validated in French and in English, and have acceptable psychometric properties. Most testing sessions took place in the participants’ home. A demographic questionnaire was used to collect basic information about the participants, such as their age, level of education and living circumstances. The questionnaire also contained items about their reading habits, both before and after the onset of their visual impairment. In addition, the Depression, Anxiety and Stress Scale (DASS 21; Lovibond and Lovibond, 1995) was used to assess the mental health of participants over the past week (Henry and Crawford, 2005) because it has good psychometric properties when used with older adults (Gloster et al., 2008).

### Vision measures

The Minnesota Low Vision Reading Test (MNRead) was used to assess the maximum reading speed (i.e., reading speed when there is no character size limit; in words per minute) and reading acuity (Mansfield et al., 1994), because it is specifically designed for low vision patients and is adapted for populations of all ages. The Mars Letter Contrast Sensitivity Test (MLCS) was used to assess contrast sensitivity, which requires participants to read letters where the contrast decreases with each letter (Arditi, 2005). Finally, the International Reading Speed Text (IReST) was used to assess reading speed (in words per minute). This test is commonly used with individuals with low vision and has been validated with younger and older adults (Trauzettel-Klosinski and Dietz, 2012; Morrice et al., 2020, 2021). Participants were encouraged to use any assistive devices that they may have to complete the task. The norms for Canadian older adults aged 60 years old and older from Morrice et al. (2021) were used. These norms only offer normative data for anglophone Canadians. Finally, participants responded to two items from the Canadian Longitudinal Study on Aging (Kirkland et al., 2015), asking “How do you characterize your eyesight (using glasses or corrected lenses if you use them)?” (excellent, very good, good, fair, poor) and “Besides glasses or contact lenses, do you use any aids or specialized equipment for persons who are blind or visually impaired” (yes/no). The second question had an optional follow-up item on which type of devices are used, to elucidate any potential effect of device access and/or use before vision rehabilitation began through professional services.

### Cognitive measures

To assess the cognitive function of participants, four tests were administered. First, the MoCA (Nasreddine et al., 2005) was used to screen the overall cognition of the participants. It was scored in both its traditional way as well as in its *blind* format(Wittich et al., 2010), because many participants were unable to complete the visual items of the test. The score of the MoCA-Blind is obtained by eliminating the visual items. A score of 26 on the MoCA and 18 on the MoCA-Blind are the cut-off scores indicating that an individual may be at risk for mild cognitive impairment. The three different versions of the MoCA (7.1, 7.2, 7.3) were assigned randomly. The normative values used for the MoCA regular version were for older adults aged 80–89 years old because the mean age and most of our participants fall into this age range (Malek-Ahmadi et al., 2015).

The Rey Auditory Verbal Learning Test (RAVLT) was used to assess the verbal learning and the verbal memory of our participants (Strauss et al., 2006). This test evaluates memory encoding, storage, and retrieval (Strauss et al., 2006). Both, the learning, delayed recall, and recognition parts were used. The RAVLT is scored for acquisition (i.e., number of words recalled in the first five trials), delayed recall (i.e., the number of words recalled after the 20-min interval) and recognition (i.e., number of words correctly identified in a 50-word list), whereby higher scores indicate better performance. The normative values used were acquired by Gale et al. (2007) in a population of older adults aged 80 to 89 years old.

A third measure was the Oral Trail Making Test parts A & B (OTMT), the auditory version of the Trail Making Test. It is designed for populations for which drawing may be a problem, such as individuals with a visual impairment (Strauss et al., 2006). It is composed of two trials, a first trial where the participant is asked to count from 1 to 25 as fast as possible, and a second trial where they are asked to count from 1 to 13 by alternating between a number and a letter in both chronological and alphabetical order. This test assesses attention, speed, and mental flexibility. The outcome measure is the time, in seconds, participants took to complete the task, whereby lower scores indicate better performance (Strauss et al., 2006). The normative values for the OTMT used in the present pilot study were acquired in a sample of older adults aged 69–90 years old.

### Hearing measures

As hearing loss is correlated with cognitive decline (Lin et al., 2013), and has been identified as the largest potentially modifiable risk factors in mid-life for dementia in late life (Livingston et al., 2020). Hearing assessment was included in the protocol to be further explored in the analyses. However, these data are only included here for descriptive purposes. First, the experimenter performed an otoscopy with the participant’s authorization. This is a visual examination of the ear canal to evaluate its integrity, using an otoscope. Both ears were examined before proceeding to the hearing test. The Canadian Digit Triplet Test (CDTT) was then used to assess speech perception in noise, using a signal-to-noise ratio threshold (Giguère et al., 2020). The outcome measure was the signal-to-noise ratio, indicating how much noise a person could tolerate while still being able to comprehend speech (more negative values indicate better tolerance of noise). Participants also complete the Hearing Handicap Inventory for the Elderly (Ventry and Weinstein, 1982) to explore self-reported situational and emotional hearing abilities.

### Procedure

All eligible individuals were identified and contacted by the admissions office at each rehabilitation center according to the eligibility criteria. If interested, their contact information was sent to the research team. Informed written consent was obtained, and participants completed the questionnaires, hearing, reading and cognitive assessment components. Participants were scheduled for their first testing session before their global initial intake exam by a professional from the rehabilitation center. All the questionnaires were administered verbally. The testing session began with the demographic questionnaire and the DASS 21. Thereafter, the participants’ vision was assessed using the MNRead and the Mars Letter Contrast Sensitivity test. Then, the MoCA and the RAVLT part 1 were performed. During the 20-min break between the RAVLT part 1 (acquisition) and part 2 (delayed recall and recognition), the participants completed the CDTT. If the participant did not need a break at this point, or if their testing session progressed at a good speed, they additionally performed the IReST and/or the OTMT, depending on how much time remained during the 20-min break. Then the RAVLT delayed recall and the RAVLT recognition were performed. The sessions lasted approximately 2 h, and the participants were encouraged to take as many breaks as they needed. The testing sessions were conducted by two experimenters (e.g., student and research assistant) to facilitate the flow of the otherwise lengthy protocol. Participants were informed that they would be contacted at a later date for eventual follow-up as part of a larger protocol that examines cognitive outcomes of vision rehabilitation (Wittich et al., 2021).

## Results

Prior to the analysis, the data were thoroughly screened for any missing or extreme values. When possible and/or suitable, the missing values were replaced by the mean of the sample or the mean of the participant (for questionnaires with multiple items). Two extreme values were removed from the data: one from the CDTT test, whereby the results indicated that the participant had an extremely severe hearing impairment that did not correspond to the communication experience with the participant and was, therefore, more likely a headphone malfunction. The second extreme value was a MoCA score of 4. Since the participant fell asleep during the MoCA, we judged that his result was not an adequate representation of his cognitive capacities. As for the other tests completed by this participant, they were included in the analysis as he was fully awake during this part of the testing, and his scores did not fall more than three SD from the group mean. Sample sizes for all available measures are provided in Table 2. Small samples (e.g., scores on the IReST) are explained by the participants’ inability to read because they had not yet received any vision rehabilitation. Missing data play a potentially important role in the assessment of cognition in persons with sensory impairment (Abraham et al., 2023); however, we did not observe any noteworthy patterns in missing cognitive measures, given that they were all chosen to accommodate the presence of visual impairment.

**Table 2:** Comparison of measures to normative values.

Measures		*n*	Scores (*M*(SD))	Normative value	*p* Value	Effect size
*Vision*						
	MNRead					
	Reading acuity	25	0.96(0,7)	0.168^1^	< 0,001^§^	0,897^a^
	Reading speed		110.4(45,5)	N/A		
	MLCS					
	Both eyes	10	Severe (0,52–1,00); *N* = 6 Moderate (1,04–1,48); *N* = 4			
	Better eye		Profound (<0,48); *N* = 1 Severe (0,52–1,00); *N* = 7 Moderate (1,04–1,48); *N* = 2		N/A	
	IReST					
	Reading speed	12	99(5,4)	190.4^2^	<0,001^§^	−1.000 ^a^
*Hearing*						
	HHIE					
	Total	38	No handicap (0–16); *N* = 30 Mild to moderate handicap (17–42); *N* = 4 Significant handicap (43+); *N* = 4			
	CDTT					
	SRT	34	−6,7(2,8)	−8,05	0,06^§^	0.338^a^
*Cognition*						
	MoCA					
	Full version	37	19,1(4,2)	25,09^5^	<0,001^¥^	−0,991^b^
	Blind version		15,8(3,1)	18.3^6^	<0,001^¥^	−0.564^b^
	RAVLT					
	Acquisition	34	33,7(11,6)	42,4^3^	0,002^¥^	−0,521^b^
	Delayed recall		5,4(3,8)	8,8^3^	<0,001^¥^	−0,637^b^
	Recognition		12,5(2,8)	13,2^3^	0,792^§^	−0,049^a^
	OTMT					
	Part A	18	9,1(3,9)	7,48^4^	0,02^¥^	0,511^b^
	Part B	17	42,7(23,4)	46,78^4^	0,092^§^	−0,415^a^

^a^Matched rank biserial correlation. ^b^Cohen’s d. ^§^Wilcoxon signed-rank test. ^¥^One sample *t*-test.

^1^Owsley et al. (1983), ^2^Morrice et al. (2021), ^3^Gale et al. (2007), ^4^Mrazik et al. (2010), ^5^Malek-Ahmadi et al. (2015), ^6^Melikyan et al. (2021).

MNRead = Minnesota low vision reading test; MLCS = Mars Letter Contrast Sensitivity Test; IReST = International Reading Speed Text; HHIE = Hearing Handicap Inventory for the Elderly; CDTT = Canadian Digit Triplet Test; SRT = Signal-to-Noise Ratio; MoCA = Montreal Cognitive Assessment; RAVLT = Rey Auditory Verbal Learning Test; OTMT = Oral Trail Making Test.

### Descriptive analysis

All participants were residents of Québec, Canada. The DASS 21 mean score was in the normal range (depression: 0–9, anxiety: 0–7, stress: 0–14). However, 15.7% of our sample showed signs of moderate depression, 13.1% showed signs of moderate anxiety, and 5.3% showed signs of moderate stress, according to the DASS-21. Most (35/38, 92%) participants were already using visual aids before the onset of their visual rehabilitation. The number of participants using various assistive devices prior to their rehabilitation is presented in Table 3. The demographic information of the participants is listed in Table 1.

**Table 3:** Assistive devices usage.

Assistive devices	Number of participants
Magnifier	35
Braille reading material	1
Large print reading material	11
Talking books	9
Recording equipment or portable note-takers	4
Closed circuit devices	6
A computer with braille, large print, or speech access	5

A participant may use more than one assistive device.

The means and the standard deviations of all test scores can be found in Table 2. Regarding the MoCA—full version, 3 (7.9%) participants had a pass score of 26 or higher, whereas, for the MoCA—blind version, 13 (34.2%) participants passed with a score of 18 or higher. A chi-squared analysis was not possible since nobody passed the MoCA-B and also failed the complete MoCA.

The mean scores on the visual, hearing, and cognitive tests, as well as the results of the t-tests for the normative comparisons to existing standard values, are listed below in Table 2. If the normality assumption was not respected, a Wilcoxon signed-rank non-parametric test was performed.

#### Vision

As would be expected for participants awaiting low vison rehabilitation, our participants had significantly lower visual acuities and reading speeds than age-matched healthy older adults (see Table 2). For the MNRead, we used data from a study by Owsley et al. (1983) as normative values to compare our sample. As their data are separated according to decade, we took the mean of the 60s, 70s and 80s age groups as the normative value for our sample. For the IReST, we used data from Morrice et al. (2021) who produced norms for adults aged 60 years old and older. There are currently no average normative values for the Letter Mars Contrast Sensitivity. However, a breakdown of our sample in terms of contrast sensitivity impairment severity in Table 2 indicates that binocular contrast sensitivity was at least moderately impaired for all participants.

#### Cognition

As for the cognitive measures, the sample performed significantly worse in both the acquisition and delayed recall parts of the RAVLT (Gale et al., 2007), as well as in the first OTMT part A (Mrazik et al., 2010), and the MoCA-Blind (Melikyan et al., 2021) in comparison to the normative values. There was no significant difference between scores from our sample and the normative data for the RAVLT recognition and OTMT part B.

## Correlation analyses

Correlations among cognition and demographic variables can be found in Table 4. Age was significantly negatively correlated with the full MoCA score (*r* = −0.365, *p* < 0.05), the RAVLT acquisition (*r* = −0.656, *p* < 0.001), the RAVLT delayed recall (*r* = −0.595, *p* < 0.001) and the RAVLT recognition (*r* = −0.461, *p* < 0.01). There was also a significant positive correlation between education and RAVLT – acquisition (*r* = 0.409, *p* < 0.05).

**Table 4:** Correlations among cognition and demographics measures.

Variables	Language	Sex	Age	DASS—S	DASS—A	DASS—D	DASS—Total
Sex	−0.227	–					
Age	−0.083	0.052	–				
DASS—S	0.216	−0.209	−0.306	–			
DASS—A	0.023	−0.006	0.048	0.330^*^	–		
DASS—D	0.109	−0.190	−0.086	0.729^***^	0.424^**^	–	
DASS—Total	0.147	−0.179	−0.154	0.862^***^	0.654^***^	0.916^***^	–
MoCA—Full	0.195	−0.103	−0.365^*^	0.089	−0.117	−0.039	−0.021
MoCA—Blind	0.141	−0.010	−0.313	0.095	−0.042	−0.032	0.009
RAVLT—Acq.	0.018	−0.081	−0.656^***^	0.326	0.120	0.135	0.246
RAVLT—Delayed	−0.053	−0.042	−0.595^***^	0.249	−0.030	−0.042	0.073
RAVLT—Recogn.	0.272	−0.107	−0.461^**^	0.273	0.128	0.059	0.188
OTMT A	0.228	0.025	0.394	0.057	0.164	0.180	0.170
OTMT B	0.220	−0.182	0.222	0.280	0.060	0.489^*^	0.395

Pearson’s correlations are used for continuous variables and Spearman’s correlations in cases where one variable is categorical; ^*^p < 0.05, ^**^p < 0.01, ^***^p < 0.001.

DASS: Depression Anxiety and Stress Scale; S: Stress; A: Anxiety; D: Depression; MoCA: Montreal Cognitive Assessment; RAVLT: Rey Auditory Verbal Learning Test; Acq.: aquisition; Recogn.: recognition; OTMT: Oral Trail Making Test.

### Cognition and vision

Pearson’s and Spearmen’s correlation coefficients between cognition and vision measures can be found in Table 5. For the vision tests, we used the MNRead (both reading acuity and reading speed), the Mars Letter Contrast sensitivity (only for both eyes and the better eye), and the vision questionnaire (both questions). For cognition, we used the MoCA (blind version only to eliminate the effect of vision), the RAVLT (acquisition, delayed recall and recognition to better understand memory processes) and the OTMT (both parts).

**Table 5:** Pearsonès correlation coefficients among cognitive and vision variables.

Variables	MoCA—Blind	RAVLT acquisition	RAVLT delayed	RAVLT recognition	OTMT A	OTMT B
MNRead—RA	0.082	−0.280	−0.126	0.133	0.542	0.281
MNRead—WPM	0.380	0.478^*^	0.239	0.301	−0.673^*^	−0.444
MLCS—OU	0.533	0.811^**^	0.476	0.872^**^	−0.470	−0.877^*^
MLCS—Better eye	0.589	0.552	0.080	0.721^*^	−0.242	−0.420
VQ—Question 1	0.028	0.083	0.298	0.050	0.042	0.309
VQ—Question 2	−0.364^*^	−0.311	−0.351^*^	−0.133	−0.018	0.420

^*^p < 0.05, ^**^p < 0.01, ^***^p < 0.001.

MNRead: Minnesota Low Vision Reading test; RA: Reading acuity; WMP: words per minute; MLCS: Mars Letter Contrast Sensitivity; OD: right eye; OS: left eye; OU: both eyes; VQ: vision questionnaire.

As for the vision and cognition correlations, the MNRead—reading speed was significantly and positively correlated with the RAVLT—acquisition score, as well as significantly and negatively correlated with OTMT part A. The binocular contrast sensitivity was positively correlated with the RAVLT—acquisition and the RAVLT—recognition, as well as negatively correlated with the OTMT part B. Additionally, contrast sensitivity in the better eye was positively correlated with the RAVLT—recognition. Finally, the second question of the vision questionnaire [“*Besides glasses or contact lenses, do you use any aids or specialized equipment for persons who are blind or visually impaired?*”] was negatively correlated with the MoCA-Blind and the RAVLT—recognition.

### Linear regressions

For every significant correlation mentioned in the previous section, a linear regression was conducted with age and education as covariates to assess if the association between these variables would remain significant. Age and education were selected because these two aspects are important to control variables in cognitive research (Rabbitt et al., 1995).

### Cognition and vision

For the MNRead—reading speed correlations, none of them remained significant after controlling for age and education.

The positive correlation between the binocular contrast sensitivity and the RAVLT—recognition remained significant (*p* = 0.018) after controlling for age and education. The other contrast sensitivity correlations did not remain significant after controlling for age and education. This result indicates that better binocular sensitivity is associated with memory, even after controlling for age and education.

For the vision questionnaire correlation, the negative correlations between the second question, the MoCA-Blind remained significant after controlling for age and education (MoCA—blind: *p* = 0.033). This indicates that older adults that are already using visual aids before their rehabilitation have better overall cognition, even after controlling for age and education. The correlation between the vision questionnaire—question 2 and the RAVLT—recognition did not remain significant after controlling for age and education.

## Discussion

The purpose of the present pilot study was to characterize the sensory and cognitive status of adults with AMD referred for visual rehabilitation. Our first objective was to assess the correlations between sensory and cognitive functions in our participants before controlling for variables known to be important in the context of cognition. The second objective was to examine which of these correlations would remain significant once established variables that influence cognition are statistically removed (i.e., age and education).

First, the association between contrast sensitivity and word recognition, as measured by the RAVLT, remained significant after controlling for age and education. This result was surprising given our limited sample size, given the reduction in statistical power by controlling for additional variables. This finding emphasizes the importance of contrast sensitivity as a variable in sensory-cognitive relationships in aging, which has previously been highlighted in the scientific literature. Globally, lower contrast sensitivity has been associated with a higher risk of mild cognitive impairment and dementia in older women, as assessed by an expanded neuropsychological tests battery and a panel of clinical experts (Ward et al., 2018). Contrast sensitivity has also been identified as a measure that is likely to detect early AD-associated changes in groups with subjective cognitive decline, amnestic mild cognitive impairment, and AD (Risacher et al., 2013). Moreover, impairments in contrast sensitivity as measured by the Pelli–Robson chart (similar in its structure to the MLCS used in the present study) are associated with a decline in global cognition as measured by the Modified Mini-Mental State Examination (Swenor et al., 2019).

Furthermore, episodic memory is an important cognitive function in sensory-cognitive aging, as it tends to decline both in normal and pathological aging. Our result is also particularly interesting in that sense, as word recognition processes are usually the ones that are the most preserved in normal aging (Perlmutter, 1979). Compared to free recall and cued recall, recognition facilitates information retrieving by presenting all the encoded information within distractors. Therefore, the recognition process helps differentiate between difficulties in encoding in comparison to difficulties retrieving information. Our results replicate studies that indicate that contrast sensitivity could be sensitive to subtle changes in cognition, specifically in the encoding process, in non-demented older adults (Risacher et al., 2013; Ward et al., 2018; Swenor et al., 2019).

One other correlation remained significant after controlling for age and education: the association between the use of visual aids and global cognition, as measured by the blind version of the MoCA. Even though our data were collected before the beginning of the low vision rehabilitation process, many participants were proactive in looking for devices that could facilitate reading and were already using various visual aids, the most popular being a magnifier. Therefore, this result may indicate that individuals already using visual aids, even before the beginning of the formal rehabilitation process, may have better global cognition, as measured by the MoCA (blind version). However, as our data are correlational, this could also mean that individuals with better baseline global cognition are more proactive, seeking out the use of visual aids even before the beginning of the formal rehabilitation process. This is also in line with the current literature, as a recent study demonstrated that the use of visual aids demonstrated a potential effect on slowing cognitive decline in a long-term care facility environment (Kwan et al., 2022).

Finally, our results corroborate findings in the scientific literature regarding the association between low vision and cognitive decline. We found that older adults with low vision have significantly poorer cognitive functions (e.g., episodic memory, processing speed, global cognition) compared to normative values. In line with previous research (Zhou et al., 2016; Zheng et al., 2018; Lee et al., 2020), these results indicate that the occurrence of vision loss in non-demented individuals is associated with poorer global cognition, memory, and executive functions.

## Mechanisms

The present results are in concordance with some postulated mechanisms explaining the link between sensory and cognitive processes. Our results could be explained by the *information degradation hypothesis*, which states that neurobiological processes, such as vision loss, diminish the strength of the perceptual signal, which negatively affect the perceptual processing and, therefore, cognitive processing (Monge and Madden, 2016). As this hypothesis does not postulate an association over time, it is possible to examine it using cross-sectional data like ours. It appears in our sample that, as compared to normative data, our low vision participants have significantly lower scores in global cognition, memory (acquisition and delayed recall), and executive functions (processing speed). Moreover, our results show that only 7.9% of our sample passed the MoCA, and 34.2% passed the MoCA-Blind. These results indicate that roughly 66% of our sample could be at risk for cognitive impairment, whereas the estimated prevalence of cognitive impairment in the Canadian population is 25% in adults 85 years old and older (Canadian Institute for Health Information, 2021).

The *sensory deprivation hypothesis* states that a persistent absence of adequate sensory input creates neuronal atrophy and later results in cognitive decline (Clay et al., 2009). The *cognitive compensation hypothesis* states that cognitive functions could be used to compensate for the visual decline, therefore monopolizing the cognitive resources and causing cognitive decline (Roberts and Allen, 2016). However, both these hypotheses require prospective data. Thus, we cannot speculate using our cross-sectional results. Our data indicate that individuals that are already using visual assistive devices before their visual rehabilitation have significantly better global cognition scores (both versions of the MoCA) and better delayed recall than those who do not already use assistive devices. As of now, our results align with only one of these proposed mechanisms, as our participants may have self-protected their cognition by initiating the rehabilitation process. Eventual prospective data are needed to assess the effect of time and rehabilitation services on cognitive functions, as planned in our larger protocol (Wittich et al., 2021).

### Vision and demographic variables

In line with previous studies (Dupuis et al., 2014; Zhou et al., 2016), our results indicated that, as age increases, global cognition and memory scores were lower. There was no significant correlation between the DASS 21 and the cognitive scores. Likely, our participants predominantly fall within the normal range of the depression, anxiety, and stress scores on the DASS 21. A recent study showed that 14% of Canadian older adults from the Canadian Longitudinal Study on Aging had symptoms of depression as measured by the Center for Epidemiological Studies Depression Scale Short Version 10 (Kuspinar et al., 2020). This is in line with our results, where 15.7% of our sample have moderate symptoms of depression. Moreover, it is noteworthy that the older portion of the Canadian population tends to present less depression, anxiety, and stress symptoms, and this is even true during the COVID-19 pandemic (Nwachukwu et al., 2020). It has been reported that older adults with depressive symptoms have lower cognitive scores than non-depressed older adults (Ganguli et al., 2006), which may explain the absence of the correlation between DASS 21 scores and any of the cognitive measure in our sample of older adults with a low proportion of depressive symptoms.

The present study is relevant to clinicians screening or assessing the cognitive status of older adults, such as neuropsychologists, because it highlights the importance of considering low vision when administering neuropsychological tests, especially to persons who have not (yet) received rehabilitation for their visual impairment. Therefore, visual ability should be screened when cognition is assessed to have an accurate assessment of cognitive functioning (Campos et al., 2019). Furthermore, the present results are important for eye care professionals that are interacting with individuals with low vision because the success of service delivery may partially depend on considering the cognitive status of the patient. Finally, the present results are interesting for researchers in the field of sensory-cognitive aging, as they highlight the importance of contrast sensitivity and the usage of visual aids for cognitive functions. Our results are in concordance with previous literature on the subject that states an association between vision, hearing and cognitive functions (Lindenberger and Baltes, 1994; Baltes and Lindenberger, 1997; Lindenberger et al., 2001; Lang et al., 2002).

### Limitations and future directions

The main limitation of this pilot study is its sample size because recruitment was interrupted due to the COVID-19 pandemic. Given the large number of measures, there is a risk of alpha error cumulation that could possibly have been controlled by adjusting the significance level (e.g., Bonferroni correction); however, such a correction would have been too strict to make the results interpretable. The protocol has now been modified to accommodate pandemic safety measures, but it remains unclear how easily pre-pandemic findings can be compared to data collected at a later point in time. Either way, more participants will be needed to strengthen the interpretation of the results. Another limitation is that the sample size is too small to control for the hearing status of our participants, even though hearing impairment has been identified as the largest potentially modifiable risk factor for dementia (Livingston et al., 2020). Moreover, only individuals with AMD were recruited for this project. Therefore, the results cannot be generalized to older adults with other ocular pathologies, such as glaucoma, diabetic retinopathy, optic neuropathy or atrophy that have each been linked to changes in cognition (Harrabi et al., 2015; Carelli et al., 2017; Lee et al., 2020). Finally, some variability in the data may be introduced by the methodological choice to test participants in their homes. Even though recruitment was facilitated because participants with visual impairment did not need to travel to a testing site, the testing conditions were not homogenous.

## Conclusion

The results of this pilot study clearly emphasize the importance of assessing the sensory abilities of older adults with low vision together with evaluating their cognitive abilities. Our participants with AMD demonstrated lower cognitive abilities than what would be expected from their sighted counterparts, even when choosing testing materials that did not rely on functional vision. A larger sample and prospective data are needed to assess the potential effect of low vision rehabilitation on cognition in older adults with AMD. The next step will be to implement the full protocol of this research project (Wittich et al., 2021), thereby expanding our understanding of sensory-cognitive aging and the role sensory rehabilitation can play in preserving cognitive function.

## Data availability statement

The raw data supporting the conclusions of this article will be made available by the authors, without undue reservation.

## Ethics statement

The studies involving human participants were reviewed and approved by Centre de recherche interdisciplinaire en réadaptation du Montréal métropolitain (CRIR #1284-1217). The patients/participants provided their written informed consent to participate in this study.

## Author contributions

GA contributed to conceptualization, formal analysis, investigation, methodology, validation, writing the original draft, and writing—review and editing. NP, AtJ, AaJ, SJ, VB, and EK contributed to conceptualization funding acquisition, formal analysis and data interpretation, investigation, methodology, resources, writing—original draft, and writing, review, and editing. WW led the conceptualization, data curation, funding acquisition, investigation, methodology, project administration, resources, supervision, validation, writing, review and editing. All authors contributed to the article and approved the submitted version.

## Funding

This study was financially supported by the *Fonds de recherche du Québec—Santé* (Programme DMLA #252235), in partnership with the *Fondation Antoine-Turmel* and the *Réseau de recherche en santé de la vision*, and the Canadian Institutes for Health Research #169713 with a patient-oriented project grant for early career investigators.

## Conflict of interest

The authors declare that the research was conducted in the absence of any commercial or financial relationships that could be construed as a potential conflict of interest.

## Publisher’s note

All claims expressed in this article are solely those of the authors and do not necessarily represent those of their affiliated organizations, or those of the publisher, the editors and the reviewers. Any product that may be evaluated in this article, or claim that may be made by its manufacturer, is not guaranteed or endorsed by the publisher.

## References

[ref1] Abraham A. G. Hong C. Deal J. A. Bettcher B. M. Pelak V. S. Gross A.. (2023). Are cognitive researchers ignoring their senses? The problem of sensory deficit in cognitive aging research. J. Am. Geriatr. Soc. doi: 10.1111/jgs.18229, PMID: 36680402

[ref2] Arditi A. (2005). Improving the design of the letter contrast sensitivity test. Investig. Ophthalmol. Vis. Sci. 46, 2225–2229. doi: 10.1167/iovs.04-1198 15914645

[ref3] Baltes P. Lindenberger U. (1997). Emergence of a powerful connection between sensory and cognitive functions across the adult life span: a new window to the study of cognitive aging? Psychol. Aging 12, 12–21. doi: 10.1037/0882-7974.12.1.12, PMID: 9100264

[ref4] Binns A. M. Bunce C. Dickinson C. Harper R. Tudor-Edwards R. Woodhouse M.. (2012). How effective is low vision service provision? A systematic review. Surv. Ophthalmol. 57, 34–65. doi: 10.1016/j.survophthal.2011.06.006, PMID: 22018676

[ref5] Bourassa K. J. Memel M. Woolverton C. Sbarra D. A. (2015). Social participation predicts cognitive functioning in aging adults over time: comparisons with physical health, depression, and physical activity. Aging Ment. Health 21, 133–146. doi: 10.1080/13607863.2015.1081152, PMID: 26327492

[ref6] Campos J. L. Höbler F. Bitton E. Labreche T. McGilton K. S. Wittich W. (2019). Screening for vision impairments in individuals with dementia living in long-term care: a scoping review. J. Alzheimers Dis. 68, 1039–1049. doi: 10.3233/JAD-181129, PMID: 30909236 PMC6484267

[ref7] Canadian Institute for Health Information (2021). Dementia in Canada: Summary. Available at: https://www.cihi.ca/en/dementia-in-canada/dementia-in-canada-summary (Accessed February 25, 2023).

[ref8] Carelli V. Morgia C. la Ross-Cisneros F. N. Sadun A. A. (2017). Optic neuropathies: the tip of the neurodegeneration iceberg. Hum. Mol. Genet. 26, R139–R150. doi: 10.1093/HMG/DDX273, PMID: 28977448 PMC5886475

[ref9] Chang L. Y. L. Lowe J. Ardiles A. Lim J. Grey A. C. Robertson K.. (2014). Alzheimer’s disease in the human eye. Clinical tests that identify ocular and visual information processing deficit as biomarkers. Alzheimers Dement. 10, 251–261. doi: 10.1016/J.JALZ.2013.06.004, PMID: 24011928

[ref10] Clay O. J. Edwards J. D. Ross L. A. Okonkwo O. Wadley V. G. Roth D. L.. (2009). Visual function and cognitive speed of processing mediate age-related decline in memory span and fluid intelligence. J. Aging Health 21, 547–566. doi: 10.1177/0898264309333326, PMID: 19436063 PMC2828155

[ref11] Corn A. L. Erin J. N. (2010). Foundations of Low Vision: Clinical and Functional Perspectives. 2nd. New York, NY: AFB Press.

[ref12] Davies-Kershaw H. R. Hackett R. A. Cadar D. Herbert A. Orrell M. Steptoe A. (2018). Vision impairment and risk of dementia: findings from the English longitudinal study of ageing. J. Am. Geriatr. Soc. 66, 1823–1829. doi: 10.1111/JGS.15456, PMID: 30098017

[ref13] Deng Y. Shi L. Lei Y. Wang D. (2016). Altered topological organization of high-level visual networks in Alzheimer’s disease and mild cognitive impairment patients. Neurosci. Lett. 630, 147–153. doi: 10.1016/J.NEULET.2016.07.043, PMID: 27461791

[ref14] Dentchev T. Milam A. H. Lee V. M.-Y. Trojanowski J. Q. Dunaief J. L. (2003). Amyloid-beta is found in drusen from some age-related macular degeneration retinas, but not in drusen from normal retinas. Mol. Vis. 9, 184–190. 12764254

[ref15] Dupuis K. Pichora-Fuller M. K. Chasteen A. L. Marchuk V. Singh G. Smith S. L. (2014). Effects of hearing and vision impairments on the Montreal cognitive assessment. Aging Neuropsychol. Cognit. 22, 413–437. doi: 10.1080/13825585.2014.968084, PMID: 25325767

[ref16] Ehrlich J. R. Swenor B. K. Zhou Y. Langa K. M. (2021). The longitudinal association of vision impairment with transitions to cognitive impairment and Dementia: Findings from The Aging, Demographics and Memory Study. J. Gerontol. A. Biol. Sci. Med. Sci. 76, 2187–93. doi: 10.1093/gerona/glab157/6290819 34061956 PMC8599065

[ref17] Gale S. D. Baxter L. Connor D. J. Herring A. Comer J. (2007). Sex differences on the Rey auditory verbal learning test and the brief visuospatial memory test–revised in the elderly: Normative data in 172 participants. J. Clin. Exp. Neuropsychol. 29, 561–567. doi: 10.1080/13803390600864760, PMID: 17564921

[ref18] Ganguli M. Du Y. Dodge H. H. Ratcliff G. G. Chang C. C. H. (2006). Depressive symptoms and cognitive decline in late life: a prospective epidemiological study. Arch. Gen. Psychiatry 63, 153–160. doi: 10.1001/ARCHPSYC.63.2.153 16461857

[ref19] Giguère C. Lagacé J. Ellaham N. N. Pichora-Fuller M. K. Goy H. Bégin C.. (2020). Development of the Canadian digit triplet test in English and French. J. Acoust. Soc. Am. 147, EL252–EL258. doi: 10.1121/10.0000825, PMID: 32237800

[ref20] Gloster A. T. Rhoades H. M. Novy D. Klotsche J. Senior A. Kunik M.. (2008). Psychometric properties of the depression anxiety and stress Scale-21 in older primary care patients. J. Affect. Disord. 110, 248–259. doi: 10.1016/J.JAD.2008.01.023, PMID: 18304648 PMC2709995

[ref21] Gordon K. Bonfanti A. Pearson V. Markowitz S. N. Jackson M. L. Small L. (2015). Comprehensive vision rehabilitation. Can. J. Ophthalmol. 50, 85–86. doi: 10.1016/j.jcjo.2014.11.009 25677290

[ref22] Government of Quebec (2022). Regulation respecting insured visual aids and related services. Available at: https://www.legisquebec.gouv.qc.ca/en/document/cr/A-29,%20r.%203 (Accessed February 25, 2023).

[ref23] Haegerstrom-Portnoy G. Schneck M. E. Brabyn J. A. Lott L. A. (2002). Development of refractive errors into old age. Optom. Vis. Sci. 79, 643–649. doi: 10.1097/00006324-200210000-00010 12395919

[ref24] Harrabi H. Kergoat M. J. Rousseau J. Boisjoly H. Schmaltz H. Moghadaszadeh S.. (2015). Age-related eye disease and cognitive function. Invest. Ophthalmol. Vis. Sci. 56, 1217–1221. doi: 10.1167/IOVS.14-15370 25650424

[ref25] Henry J. D. Crawford J. R. (2005). The short-form version of the depression anxiety stress scales (DASS-21): construct validity and normative data in a large non-clinical sample. Br. J. Clin. Psychol. 44, 227–239. doi: 10.1348/014466505X29657 16004657

[ref26] Hong T. Mitchell P. Burlutsky G. Liew G. Wang J. J. (2016). Visual impairment, hearing loss and cognitive function in an older population: longitudinal findings from the blue mountains eye study. PLoS One 11:e0147646. doi: 10.1371/journal.pone.0147646, PMID: 26808979 PMC4726694

[ref27] Ikram M. K. Cheung C. Y. Wong T. Y. Chen C. P. L. H. (2012). Retinal pathology as biomarker for cognitive impairment and Alzheimer’s disease. J. Neurol. Neurosurg. Psychiatry 83, 917–922. doi: 10.1136/JNNP-2011-301628 22733082

[ref28] Kiely K. M. Anstey K. J. (2015). “Common Cause Theory in Aging,” in Encyclopedia of Geropsychology. ed. Pachana N. (Singapore: Springer) (2015). 1–11.

[ref29] Kirby E. Bandelow S. Hogervorst E. (2010). Visual impairment in Alzheimer’s disease: a critical review. J. Alzheimers Dis. 21, 15–34. doi: 10.3233/JAD-2010-080785 20182034

[ref30] Kirkland S. A. Griffith L. E. Menec V. Wister A. Payette H. Wolfson C.. (2015). Mining a unique Canadian resource: the Canadian longitudinal study on aging. Can. J. Aging 34, 366–377. doi: 10.1017/S071498081500029X, PMID: 26300192

[ref31] Kuspinar A. Verschoor C. P. Beauchamp M. K. Dushoff J. Ma J. Amster E.. (2020). Modifiable factors related to life-space mobility in community-dwelling older adults: results from the Canadian longitudinal study on aging. BMC Geriatr. 20, 1–12. doi: 10.1186/S12877-020-1431-5/FIGURES/2 PMC6995110 32005107

[ref32] Kwan R. Y. C. Kwan C. W. Kor P. P. K. Chi I. (2022). Cognitive decline, sensory impairment, and the use of audio-visual aids by long-term care facility residents. BMC Geriatr. 22:216. doi: 10.1186/s12877-022-02895-x, PMID: 35296238 PMC8928635

[ref33] Lang R. Rieckmann N. Baltes M. (2002). Adapting to aging losses. Do resources facilitate strategies of selection compensation and optimization in everyday functioning? Journals of gerontology B. Psychol. Sci. 57B, 501–509. doi: 10.1093/geronb/57.6.P501 12426432

[ref34] Lee A. G. Martin C. O. (2004). Neuro-ophthalmic findings in the visual variant of Alzheimer’s disease. Ophthalmology 111, 376–380. doi: 10.1016/S0161-6420(03)00732-2, PMID: 15019393

[ref35] Lee S. S. Y. Wood J. M. Black A. A. (2020). Impact of glaucoma on executive function and visual search. Ophthalmic Physiol. Opt. 40, 333–342. doi: 10.1111/OPO.12679, PMID: 32189400

[ref36] Lin F. Yaffe K. Xia J. Xue Q.-L. Harris T. B. Purchase-Helzner E.. (2013). Hearing loss and cognitive decline in older adults. JAMA Intern. Med. 173, 293–299. doi: 10.1001/jamainternmed.2013.1868, PMID: 23337978 PMC3869227

[ref37] Lindenberger U. Baltes P. (1994). Sensory functioning and intelligence in old age: a strong connection. Psychol. Aging 9, 339–355. doi: 10.1037/0882-7974.9.3.339, PMID: 7999320

[ref38] Lindenberger U. Scherer H. Baltes P. (2001). The strong connection between sensory and cognitive performance in old age: not due to sensory acuity reductions operating during cognitive assessment. Psychol. Aging 16, 196–205. doi: 10.1037/0882-7974.16.2.196, PMID: 11405308

[ref39] Livingston G. Huntley J. Sommerlad A. Ames D. Ballard C. Banerjee S.. (2020). Dementia prevention, intervention, and care: 2020 report of the lancet commission. Lancet 396, 413–446. doi: 10.1016/S0140-6736(20)30367-6, PMID: 32738937 PMC7392084

[ref40] Lovibond P. F. Lovibond S. H. (1995). The structure of negative emotional states: comparison of the depression anxiety stress scales (DASS) with the Beck depression and anxiety inventories. Behav. Res. Ther. 33, 335–343. doi: 10.1016/0005-7967(94)00075-u, PMID: 7726811

[ref41] Malek-Ahmadi M. Powell J. J. Belden C. M. Oconnor K. Evans L. Coon D. W.. (2015). Age- and education-adjusted normative data for the Montreal cognitive assessment (MoCA) in older adults age. Neuropsychol. Dev. Cogn. B Aging Neuropsychol. Cogn. 22, 755–761. doi: 10.1080/13825585.2015.1041449, PMID: 25942388

[ref42] Mansfield J. S. Legge G. E. Luebker A. Cunningham K. (1994). MNRead Acuity Charts; Continuous-text Reading-acuity Charts for Normal and Low Vision. Long Island City NY: Distributed by Lighthouse Low Vision Products.

[ref43] Melikyan Z. A. Malek-Ahmadi M. O’Connor K. Atri A. Kawas C. H. Corrada M. M. (2021). Norms and equivalences for MoCA-30, MoCA-22, and MMSE in the oldest-old. Aging Clin. Exp. Res. 33, 3303–3311. doi: 10.1007/S40520-021-01886-Z/FIGURES/1, PMID: 34050916 PMC8668848

[ref44] Monge Z. A. Madden D. J. (2016). Linking cognitive and visual perceptual decline in healthy aging: the information degradation hypothesis. Neurosci. Biobehav. Rev. 69, 166–173. doi: 10.1016/J.NEUBIOREV.2016.07.031, PMID: 27484869 PMC5030166

[ref45] Morrice E. Hughes J. Stark Z. Wittich W. Johnson A. (2020). Validation of the international Reading speed texts in a Canadian sample. Optom. Vis. Sci. 97, 509–517. doi: 10.1097/OPX.0000000000001538, PMID: 32697558

[ref46] Morrice E. Soldano V. Addona C. Murphy C. E. Johnson A. P. (2021). Validation of the international Reading speed texts in a sample of older (60+) Canadian adults. Optom. Vis. Sci. 98, 971–975. doi: 10.1097/OPX.0000000000001746, PMID: 34460456

[ref47] Mrazik M. Millis S. Drane D. L. (2010). The oral trail making test: effects of age and concurrent validity. Arch. Clin. Neuropsychol. 25, 236–243. doi: 10.1093/arclin/acq006, PMID: 20197294 PMC2858599

[ref48] Nagarajan N. Assi L. Varadaraj V. Motaghi M. Sun Y. Couser E.. (2022). Vision impairment and cognitive decline among older adults: a systematic review. BMJ Open 12:e047929. doi: 10.1136/bmjopen-2020-047929, PMID: 34992100 PMC8739068

[ref49] Nasreddine Z. S. Phillips N. A. Bedirian V. Charbonneau S. Whitehead V. Collin I.. (2005). The Montreal cognitive assessment, MoCA: a brief screening tool for mild cognitive impairment. J. Am. Geriatr. Soc. 53, 695–699. doi: 10.1111/j.1532-5415.2005.53221.x, PMID: 15817019

[ref50] Nwachukwu I. Nkire N. Shalaby R. Hrabok M. Vuong W. Gusnowski A.. (2020). COVID-19 pandemic: age-related differences in measures of stress, anxiety and depression in Canada. Int. J. Environ. Res. Public Health 2020 17:6366. doi: 10.3390/IJERPH17176366, PMID: 32882922 PMC7503671

[ref51] Owsley C. Sekuler R. Siemsen D. (1983). Contrast sensitivity throughout adulthood. Vis. Res. 23, 689–699. doi: 10.1016/0042-6989(83)90210-9 6613011

[ref52] Park D. Gutchess A. (2016). The cognitive neuroscience of aging and culture. Curr. Dir. Psychol. Sci. 15, 105–108. doi: 10.1111/j.0963-7214.2006.00416.x

[ref53] Perlmutter M. (1979). Age differences in adults’ free recall, cued recall, and recognition. J. Gerontol. 34, 533–539. doi: 10.1093/GERONJ/34.4.533 448044

[ref54] Pierscionek B. K. Weale R. A. (1995). The optics of the eye-lens and lenticular senescence. A review. Doc. Ophthalmol. 89, 321–335. doi: 10.1007/BF01203708, PMID: 7493534

[ref55] Rabbitt P. Donlan C. Watson P. McInnes L. Bent N. (1995). Unique and interactive effects of depression, age, socioeconomic advantage, and gender on cognitive performance of normal healthy older people. Psychol. Aging 10, 307–313. doi: 10.1037//0882-7974.10.3.307, PMID: 8527052

[ref56] Ratnayaka J. A. Serpell L. C. Lotery A. J. (2015). Dementia of the eye: the role of amyloid beta in retinal degeneration. Eye 2015 8, 1013–1026. doi: 10.1038/eye.2015.100,29,1013,1026, PMID: 26088679 PMC4541342

[ref57] Risacher S. L. WuDunn D. Pepin S. M. MaGee T. R. McDonald B. C. Flashman L. A.. (2013). Visual contrast sensitivity in Alzheimer’s disease, mild cognitive impairment, and older adults with cognitive complaints. Neurobiol. Aging 34, 1133–1144. doi: 10.1016/J.NEUROBIOLAGING.2012.08.007, PMID: 23084085 PMC3545045

[ref58] Roberts K. L. Allen H. A. (2016). Perception and cognition in the ageing brain: a brief review of the short- and long-term links between perceptual and cognitive decline. Front. Aging Neurosci. 8, 1–7. doi: 10.3389/fnagi.2016.00039, PMID: 26973514 PMC4772631

[ref59] Rogers M. A. M. Langa K. M. (2010). Untreated poor vision: a contributing factor to late-life dementia. Am. J. Epidemiol. 171, 728–735. doi: 10.1093/aje/kwp453, PMID: 20150357 PMC2842219

[ref60] Spierer O. Fischer N. Barak A. Belkin M. (2016). Correlation between vision and cognitive function in the elderly: a cross-sectional study. Medicine (United States) 95, e2423–e2425. doi: 10.1097/MD.0000000000002423, PMID: 26817872 PMC4998246

[ref61] Strauss E. Sherman E. Spreen O. (2006). A compendium of Neuropsychological Tests: Administration, Norms, and Commentary. Oxford, England.

[ref62] Swenor B. K. Lesche S. Varadaraj V. Ramulu P. Y. (2016). Short and sustained reading speeds in AMD patients: an interaction with cognitive status. Invest. Ophthalmol. Vis. Sci. 57:1947.

[ref63] Swenor B. K. Wang J. Varadaraj V. Rosano C. Yaffe K. Albert M.. (2019). Vision impairment and cognitive outcomes in older adults: the health ABC study. J. Gerontol. A Biol. Sci. Med. Sci. 74, 1454–1460. doi: 10.1093/gerona/gly244, PMID: 30358809 PMC6696724

[ref64] Trauzettel-Klosinski S. Dietz K. (2012). Standardized assessment of reading performance: the new international reading speed texts IReST. Invest. Ophthalmol. Vis. Sci. 53, 5452–5461. doi: 10.1167/iovs.11-8284, PMID: 22661485

[ref65] Ventry I. M. Weinstein B. E. (1982). The hearing handicap inventory for the elderly: a new tool. Ear Hear. 3, 128–134. doi: 10.1097/00003446-198205000-00006 7095321

[ref66] Ward M. E. Gelfand J. M. Lui L. Y. Ou Y. Green A. J. Stone K.. (2018). Reduced contrast sensitivity among older women is associated with increased risk of cognitive impairment. Ann. Neurol. 83, 730–738. doi: 10.1002/ANA.25196, PMID: 29518257 PMC5947874

[ref67] Whitson H. E. Woolson S. Olsen M. Massof R. Ferguson S. M. Muir K. W.. (2020). Cognitive impairment among veterans in outpatient vision rehabilitation. Optom. Vis. Sci. 97, 462–469. doi: 10.1097/OPX.0000000000001522, PMID: 32511169 PMC7291825

[ref68] Wittich W. Al-Yawer F. Phillips N. (2019). Visual acuity and contrast sensitivity at various stages of cognitive impairment in the COMPASS-ND study. Invest. Ophthalmol. Vis. Sci. 60:5913.

[ref69] Wittich W. Gagné J. (2016). “Perceptual aspects of gerotechnology” in Gerontechnology: Research, Practice, and Principles in the Field of Technology and Aging. ed. Kwon S. (New York NY: Springer Publishing Co), 13–34.

[ref70] Wittich W. Phillips N. Nasreddine Z. S. Chertkow H. (2010). Sensitivity and specificity of the Montreal cognitive assessment modified for individuals who are visually impaired. J. Vis. Impair Blind 104, 360–368. doi: 10.1177/0145482X1010400606

[ref71] Wittich W. Pichora-Fuller M. K. Johnson A. Joubert S. Kehayia E. Bachir V.. (2021). Effect of reading rehabilitation for age-related macular degeneration on cognitive functioning: protocol for a nonrandomized pre-post intervention study. JMIR Res. Protoc. 10:e19931. doi: 10.2196/19931, PMID: 33704074 PMC7995070

[ref72] Wong W. L. Su X. Li X. Cheung C. M. G. Klein R. Cheng C.-Y.. (2014). Global prevalence of age-related macular degeneration and disease burden projection for 2020 and 2040: a systematic review and meta-analysis. Lancet Glob. Health 2, e106–e116. doi: 10.1016/S2214-109X(13)70145-1, PMID: 25104651

[ref73] World Health Organization (2017). Global action plan on the public health response to dementia 2017–2025. Geneva, World Health Organization.

[ref74] World Health Organization (2019). World Report on Vision. Available at https://www.who.int/publications-detail-redirect/9789241516570 (Accessed August 3, 2021).

[ref75] Zheng D. D. Swenor B. K. Christ S. L. West S. K. Lam B. L. Lee D. J. (2018). Longitudinal associations between visual impairment and cognitive functioning. JAMA Ophthalmol. 136, 989–995. doi: 10.1001/jamaophthalmol.2018.2493, PMID: 29955805 PMC6142982

[ref76] Zhou L. X. Sun C. L. Wei L. J. Gu Z. M. Lv L. Dang Y. (2016). Lower cognitive function in patients with age-related macular degeneration: a meta-analysis. Clin. Interv. Aging 11, 215–223. doi: 10.2147/CIA.S102213, PMID: 26966358 PMC4771401

